# Synthetic Approaches and Biological Activities of 4-Hydroxycoumarin Derivatives

**DOI:** 10.3390/molecules14114790

**Published:** 2009-11-23

**Authors:** Jae-Chul Jung, Oee-Sook Park

**Affiliations:** 1 Ewha Global Challenge, BK21 and Medical Research Institute, School of Medicine, Ewha Womans University, Seoul 158–710, Korea; E-Mail: jcjung10@yahoo.co.kr (J.-C.J.); 2 Department of Chemistry, Institute for Basic Sciences, College of Natural Sciences, Chungbuk National University, Cheongju 361–763, Chungbuk, Korea

**Keywords:** 4-hydroxycoumarin, anticoagulant, rodenticide, ring cyclization, tetralone, coupling reaction

## Abstract

The main purpose of this review is to summarize recent chemical syntheses and structural modifications of 4-hydroxycoumarin and its derivatives, of interest due to their characteristic conjugated molecular architecture and biological activities.

## 1. Introduction

**Figure 1:**
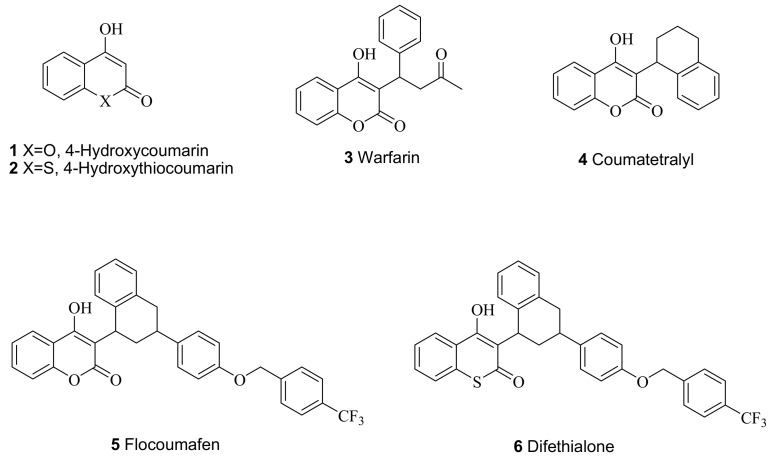
Structures of the 4-hydroxycoumarin (**1**), 4-hydroxythiocoumarin (**2**), and derivatives **3-6**.

4-Hydroxycoumarins (2*H*-1-benzopyran-2-ones, Figure 1) have evoked a great deal of interest due to their biological properties and characteristic conjugated molecular architecture. Many of them display important pharmacological effects, including analgesic [1], anti-arthritis [2], anti-inflammatory [3], anti-pyretic [4], anti-bacterial [5], anti-viral [6], and anti-cancer [7] properties. 4-Hydroxy-coumarin and its derivatives have been effectively used as anticoagulants for the treatment of disorders in which there is excessive or undesirable clotting, such as thrombophlebitis [8], pulmonary embolism [9], and certain cardiac conditions [10]. A number of comparative pharmacological investigations of the 4-hydroxycoumarin derivatives have shown good anticoagulant activity combined with low side effects and little toxicity [11]. 

Nowadays 4-hydroxycoumarin and its derivatives are widely used anticoagulant rodenticides as well as antithrombotic agents [12]. The 4-hydroxycoumarin anticoagulants are antagonists of vitamin K and their target is vitamin K 2,3-epoxide reductase in the liver microsomes. Finally, they are also useful key intermediates for many industrial products such as dyes [13] and liquid crystals [14].

The chemical synthesis, structural modification, and a wide variety of biological activities of 4-hydroxycoumarins have been reported in many papers [15,16,17]. The goal of this review is to summarize recent synthetic approaches to 4-hydroxycoumarin derivatives and their biological activities.

## 2. Results and Discussion

**Scheme 1:**
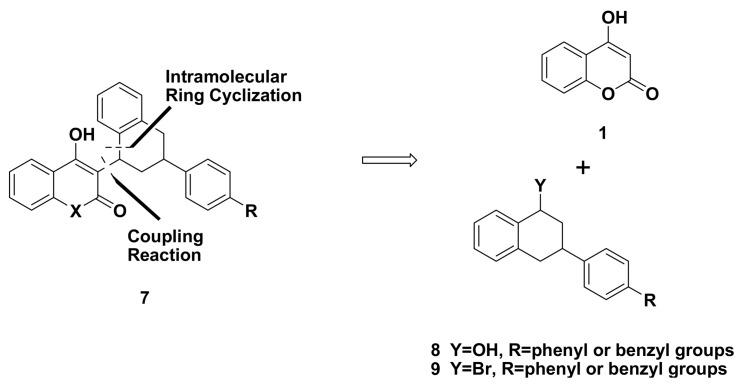
Retrosynthetic analysis of the 4-hydroxycoumarin derivatives.

The 2*H*-1-benzopyran-2-one and tetrahydronaphthalen-1-ols skeletons are essential structural features in the second generation rodenticide 4-hydroxycoumarin derivatives. They have traditionally been coupled in acidic media. Although several improved condensation reactions of 4-hydroxy-coumarin (**1**) with compounds **8-9** using Bronsted-Lowry acids (HCl, H_2_SO_4_, *p*-TsOH) have been reported [18,19,20,21], these reactions led to preferential dehydrohalogenation, resulting in low yields. Thus, an efficient coupling condition was required to obtain better yield. Scheme 1 shows a representative retrosynthetic approach for this class of molecules. The tetrahydronaphthalen-1-ol **8** was coupled with 4-hydroxycoumarin (**1**) or 4-hydroxythiocoumarin (**2**) to generate the target 2*H*-1-benzopyran-2-one or 2*H*-1-benzothiopyran-2-one.

**Scheme 2:**
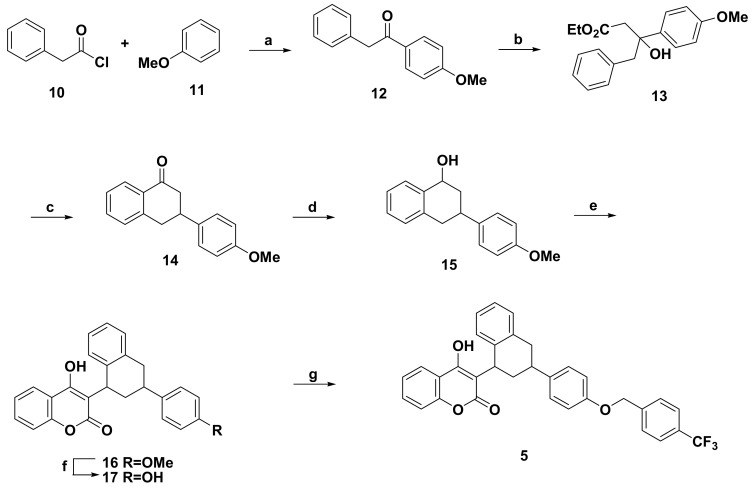
Synthesis of flocoumafen (**5**).

Our previous synthesis of flocoumafen (**5**) [18,19] is summarized in Scheme 2. Its main reactions were Friedel-Crafts acylation, Reformatsky reaction, and dehydration. Commercially available phenylacetyl chloride (**10**) was condensed with anisole (**11**) in the presence of AlCl_3_ to afford ketone **12**, which was treated with ethyl bromoacetate to give hydroxyl ethyl ester **13** in 86% yield over two steps. Subsequent dehydroxylation of ethyl ester **13** was accomplished with triethylsilane and boron trifluoride to give the corresponding ester, which was smoothly hydrolyzed under basic conditions and cyclized using polyphosphoric acid to yield tetralone **14** in three steps. Reduction of tetralone **14** with sodium borohydride afforded secondary alcohol **15**, which was then coupled with 4-hydroxycoumarin in the presence of *p*-toluenesulfonic acid to give compound **16**. Demethylation of compound **16** was performed with hyrobromic acid in acetic acid to give phenol **17**. Phenol **17** was *O*-alkylated with freshly prepared 3-(trifluoromethyl)benzyl bromide in sodium hydride/THF to generate flocoumafen (**5**) in good yield (overall yield was 25% in eight steps).

**Scheme 3:**
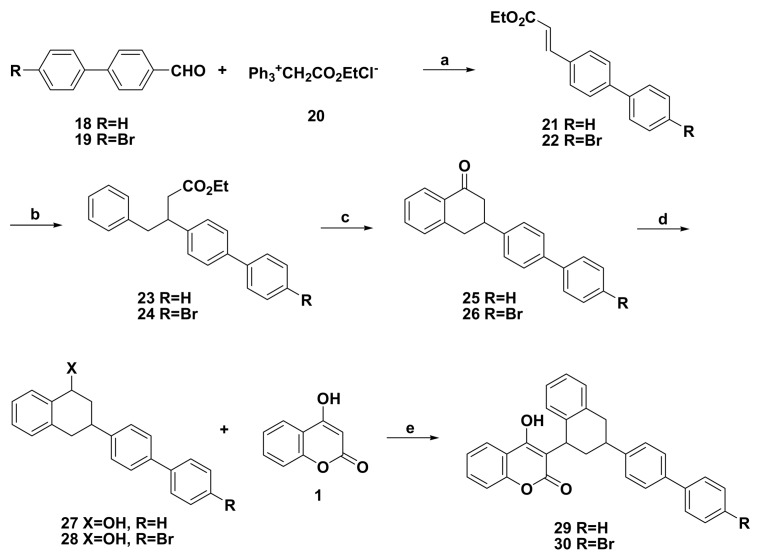
Synthesis of diphenacoum (**29**) and brodifacoum (**30**).

The Ferreira group [20] has developed to a new protocol for the synthesis of diphenacoum (**29**) and brodifacoum (**30**). The key step involves the stereospecific formation of one of the crucial bonds in the molecular backbone using asymmetric organocopper 1,4-addition to chiral imides. Wittig condensation of freshly prepared aldehydes **18**, **19**, and (carbethoxy)triphenylphosphonium chloride (**20**) in the presence of sodium methoxide in DMF gave the biphenyl esters **21**, **22** in 92% and 87% yield, respectively. Organocopper methodology was then successfully applied to the synthesis of butanoate. 1,4-Michael addition with compounds **21**, **22** and BnCu-TMEDA complex in the presence of TMS-Cl generated **23**, **24** in 84% and 81% yield, respectively, and then subsequent ring cyclization by using AlCl_3_ in toluene to give tetralones **25** and **26** in 88% and 86% yield, respectively. The coupling reaction between 4-hydroxycoumarin **1** and secondary alcohol **27** or brominated compound **28** under an HCl atmosphere at 160 °C 30 min provided approximately equal quantities of the *cis* and *trans* isomers of 4-hydroxycoumarin derivatives **29** and **30** in 78% and 74% yield, respectively (Scheme 3).

**Scheme 4:**
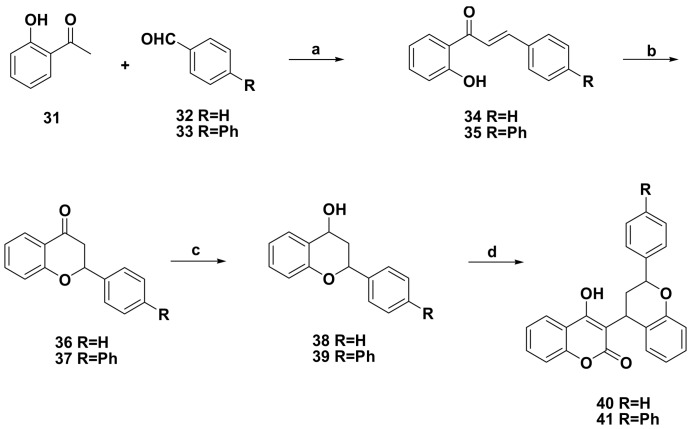
Synthesis of novel diphenacoum analogues.

The Yang group [21] reported the synthesis of novel diphenacoum analogues using base-catalyzed aldol condensation, ring cyclization, and coupling reactions. 2-Hydroxyacetophenone (**31**) was treated with aldehydes **32** or **33** under base-catalyzed aldol reaction conditions to produce ketones **34**, **35**, which were readily cyclized by phosphoric acid in ethanol to give 2-biphenylchroman-4-ones **36** and **37**, respectively. Reduction of **36** and **37** with sodium borohydride in methanol gave quantitative yields of the corresponding alcohols **38** and **39**, which were then condensed with 4-hydroxycoumarin (**1**) in 1,2-dichloroethane in the presence of a catalytic amounts of *p*-toluenesulfonic acid, to yield the target compounds **40** and **41**, respectively (Scheme 4). 

**Scheme 5:**
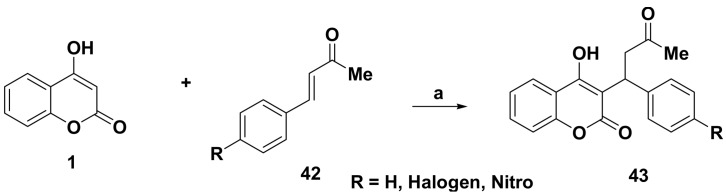
Danchev synthesis of 4-hydroxcoumarin derivatives.

The Danchev group [22] reported a synthesis of 4-hydroxcoumarin derivatives and their anticoagulant activities. Their method involves a condensation reaction of 4-hydroxcoumarin (**1**) with unsaturated ketone **42** or substituted aromatic aldehydes **32-33** (Scheme 5). Warfarin type compound **43** showed similar anticoagulant effect as coumachlor or warfarin *in vivo*, while its acute toxicity was higher than that of woumachlor. Among their 4-hydroxcoumarin derivatives 3,3’-(4-chlorophenylmethylene)-bis-(4-hydroxy-2H-1-benzopyran-2-one), with low toxicity, is a prospective lead compound. 

**Scheme 6:**
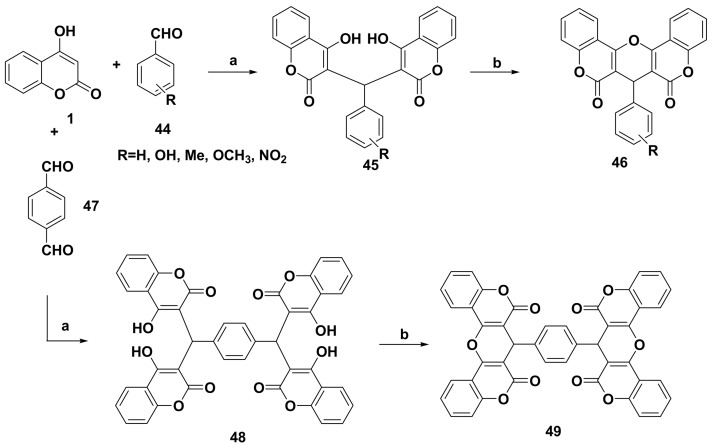
Synthesis of benzopyranodicoumarins **46** and **49**.

The Hamdi group [23] prepared benzopyranodicoumarins **46** and **49** and evaluated their antioxidative and antibacterial activities. Aromatic aldehydes **44** containing different groups in the *ortho*-, *meta-* or *para-* positions was condensed with 4-hydroxycoumarin (**1**) in ethanol and acetic acid to generate substituted 3,3’-arylidenebis-4-hydroxycoumarins **45** and tetrakis-4-hydroxycoumarin derivatives **48.** Heating of compounds **45** and **48** in acetic anhydride transformed them into benzopyranodicoumarins **46** and **49** (Scheme 6).

**Scheme 7:**
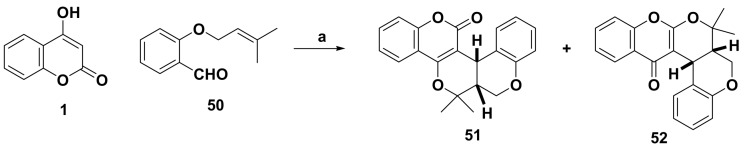
Synthesis of pyrano[3-2c]coumarins.

On the other hand, the Raghunathan group [24] successfully established a pyrano[3-2c]coumarin framework using microwave accelerated intramolecular domino Knoevenagel-hetero Diels-Alder reactions. 4-Hydroxycoumarin (**1**) was treated with 2-(3-methyl-2-butenyloxy)benzaldehyde under microwave irradiation in ethanol for 15 s to give a 97:3 ratio of pyrano[3-2c]coumarin **51** and pyrano[3-2c]chromene derivative **52** in good yield (Scheme 7). This methodology is very useful, providing an easy access to the pyrano[3-2c]coumarin skeleton found in many natural products.

**Scheme 8:**
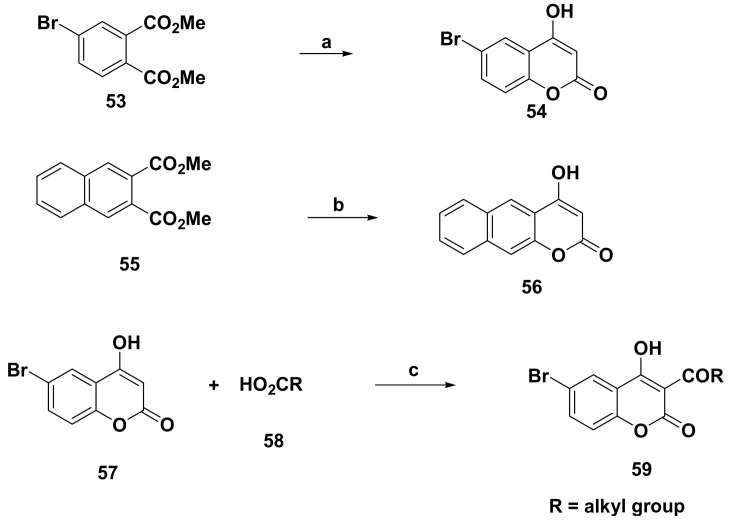
Synthesis of substituted 4-hydroxycoumarins **54** and **56** and dicoumairn **59**.

The Trkovnik group [25] synthesized substituted 4-hydroxycoumarins **54** and **56** from compounds **53** and **55** according to a modified Pauly and Lockemann method [26], respectively. They also prepared dicoumairn type moiety **59** in good yield (Scheme 8).

**Scheme 9:**
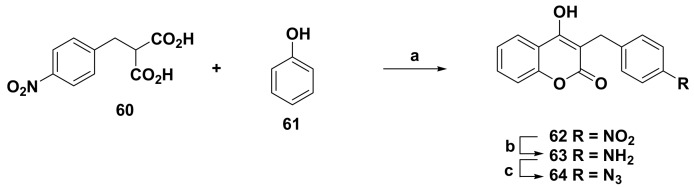
Synthesis of 3-(*p*-azido, amino, and nitrobenzyl)-4-hydroxycoumarins **62-64**.

The Swenson group [27] recently published a unique procedure for preparation of 3-(*p*-azido, amino, and nitrobenzyl)-4-hydroxycoumarins **62-64** by the reaction of freshly prepared benzyl malonic acid **60** and phenol (**61**) in the presence of phosphorus trichloride and zinc chloride (Scheme 9). They also prepared various radiolabeled 3-substituted 4-hydroxycoumarin derivatives using commercially available [U-^14^C] phenol or [U-^3^H] in order to elucidate the binding photoaffinity. Compounds **62-64** serve as effective competitive inhibitors of the dicoumarol sensitive NADPH quinone reductase from rat liver [28,29].

**Scheme 10:**
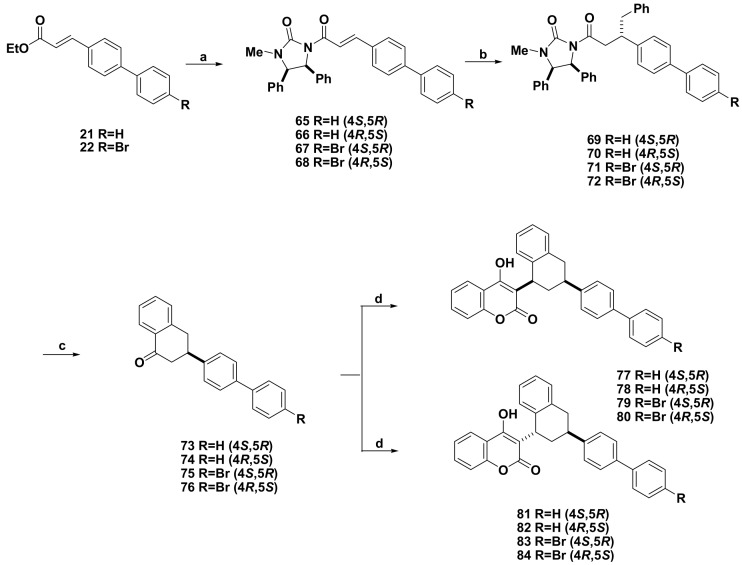
Stereo- and enantioselective synthesis of diphenacoum (**29**) and brodifacoum (**30**).

Recently, stereoselective synthesis has been an important topic in the synthesis of biologically active substances. Efficient asymmetric syntheses of 4-hydroxycoumarin derivatives were demonstrated by two groups. The Ferreira group [30] firstly reported a highly stereo- and enantio- selective synthesis of diphenacoum (**29**) and brodifacoum (**30**). The key step involves a stereospecific 1,4-Michael addition using a chiral auxiliary and intramolecular ring cyclization. The esters **21**, **22** were transformed into acid chlorides through hydrolysis and chlorination with KOH and SOCl_2_, and subsequently reacted with the lithium anion of the chiral auxiliary to afford the chiral imides **65-68** in 72%, 74%, 73%, and 70% yield, respectively, for three steps. Asymmetric 1,4-Michael addition of imides **65-68** was accomplished with Bn-Cu-TMEDA complex in the presence of Bu_2_BOTf to give diastereoisomeric ketones **69-72**, which were effectively cyclized using trifluoromethanesulfonic acid to generate chiral tetralones **73-76** with 99% optical purity in 85%, 84%, 79%, and 80% yield, respectively. Reduction of tetralones **73-76** with sodium borohydride afforded the corresponding *cis* benzyl alcohols, which were condensed with 4-hydroxcoumarin (**1**) to give *cis*/*trans* diphenacoum and brodifacoum, respectively (Scheme 10). The stereoisomeric mixtures were readily separated by flash column chromatography. The configuration was established using spectroscopic analysis.

**Scheme 11:**
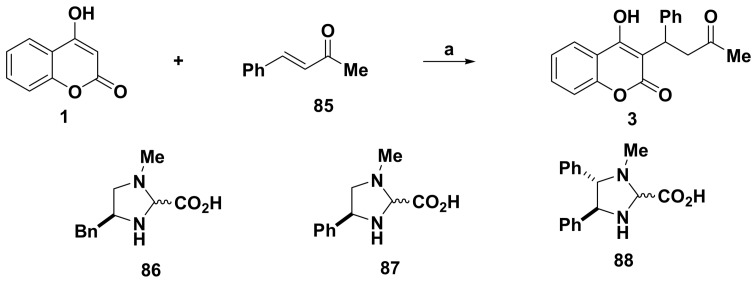
Organocatalytic asymmetric 1,4-Michael addition of 4-hydroxycoumarin and α,β–unsaturated ketones.

The Jorgensen group [31] reported a highly economical organocatalytic asymmetric 1,4-Michael addition of 4-hydroxycoumarin and α,β–unsaturated ketones to generate the widely used anticoagulants warfarin (Coumadin) and some related important compounds (Scheme 11). They attempted enantioselective 1,4-Michael addition by using well known benzylideneacetophenone (**85**) and 4-hydroxycoumarin (**1**) in the presence of optically active imidazolidine catalysts **86-88** to afford enantiopure warfarin moieties with 47 to 82% ee values in 22 to 96% yields. This asymmetric one-step method could be useful for the formation of a number of biologically active compounds.

**Scheme 12:**
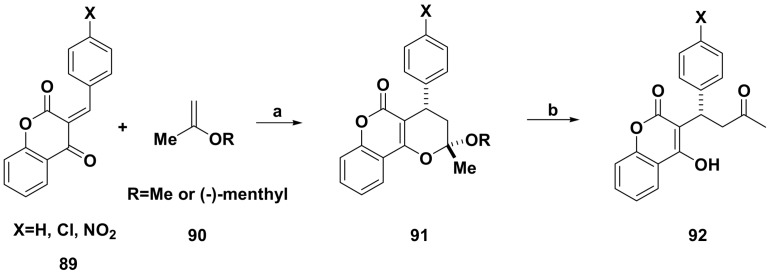
Asymmetric two-step synthesis of non-racemic coumarin anticoagulants.

The Tagliapietra group [32] developed an asymmetric two-step synthesis of non-racemic coumarin anticoagulants such like warfarin, coumachlor, and acenocoumarol through one-pot-three-component tandem Knoevenagel-hetero Diels-Alder cycloaddition reactions between *in situ* generated 3-arylidene-2,4-chromanediones **89** and isopropenyl ether **90** derived from (-)-menthol in 61% yield with 95% ee for (*S*)-warfarin, 56% yield with 93% ee for (*S*)-coumachlor, and 59% yield with 95% ee for (*S*)-acenocoumarol (Scheme 12). Knoevenagel adducts **91** were treated with 3N-HCl in the presence of SiO_2_ or TFA:H_2_O (19:1, v/v) as a reaction promotor to get the final asymmetric products **92** in nearly quantitative yields.

## 3. Biological Activity

A large number of 4-hydroxycoumarins and their derivatives have been synthesized and evaluated for their ability to play a positive role in the prevention of human and animal diseases. Various pharmacological activities for the representative compounds have been described in the literature (Table 1). Most 4-hydroxycoumarin derivatives showed representative anticoagulant effects, while compounds **3**, **5**, **30**, **40**, **46**, and **64** exhibit a wide variety of activities including antiarthritis, anti-inflammatory, anticancer, antithrombosis, teratogenic, antibacterial, and photoaffinity effects. 

Anticoagulant is the most prominent among many intriguing pharmacological effects observed for many 4-hydroxycoumarin derivatives. In particular warfarin, as first generation anticoagulant, and compounds like brodifacoum, bromadiolone, chlorophacinone, difenacoum, coumatetralyl, flocoumafen, and difethialone are effective anticoagulant rodenticides. The second generation anticoagulant rodenticides, difenacoum (**29**) and brodifacoum (**30**) showed a plasma elimination half-life of 91.7 days, while liver elimination half-lives varied from 15.8 days for coumatetralyl (**4**) to 307.4 days for brodifacoum. In general, the elimination half-lives in plasma for first-generation rodenticides were shorter than those for second-generation rodenticides. These results revealed that the so-called superwarfarins such as difenacoum (**29**), brodifacoum (**30**), flocoumafen (**5**), and difethialone (**6**) showed higher anticoagulant effects than warfarin [48]. The biological activities of compounds **40** and **41** indicate potent vitamin K 2,3-epoxide reductase (VKOR) inhibition effects with IC_50_ values of 0.4 μM, comparable with warfarin. Compound **40** was shown to be 2.5-fold more potent than warfarin, while compound **41** exhibited 10 times less activity than warfarin. These biological results imply that the hydrogen bonding has a major effect on enzyme site binding [21]. 4-Hydroxycoumarin derivatives **43** showed favorable anticoagulant effect compared with warfarin; especially a chlorine at the *para*-position in the aromatic ring resulted in potent anticoagulant activities compared to nitro or other halogen substituents at the *para*-position [22,49]. The dicoumarol related compounds **48-49** were evaluated for antimicrobial, antioxidant activities using MIC tests and radical scavenging activities. Also, compounds **48** and **49** showed favorable antimicrobial activity compared to warfarin and similar effects to each other for the antioxidant activity. In addition, the coumarol moiety showed more potent activity than a benzopyranocoumarol moiety due to the stable configuration and favorable binding activity through hydrogen bonding in the enzyme binding site [22]. 6-Bromo-4-hydroxycoumarin (**54**) and 4-hydroxy-6,7-benzocoumarin (**56**) exhibited significant anticoagulant effect with a rapid short duration. Especially 3,3’-alkylidene bis-6-bromo-4-hydroxycoumarin derivatives showed a potent anticoagulant activity *in vitro* [25].

A 4-hydroxycoumarin containing an azidobenzyl group at the 3-position − 3-(*p*-azidobenzyl)-4-hydroxycoumarin (**64**)—showed an inhibition constant of 6.6 × 10^-8^ M, a value comparable to that observed for dicoumarol (1.7 × 10^-9^ M), but significantly lower than that for warfarin (3.5 × 10^-5^ M). This result implies that compound **64** can be an effective photoaffinity probe in the identification of other proteins associated with the vitamin K-dependent carboxylation system that are similarly inhibited by 4-hydroxycoumarin derivatives [46].

## 4. Conclusions

The chemical syntheses and structural modifications of 4-hydroxycoumarin and its derivatives are of interest due to their biological activities and characteristic conjugated molecular architecture. This review summarized the recent synthetic approaches to 4-hydroxycoumarin derivatives and the current state of research into their biological activities.

## Figures and Tables

*Reagents and conditions*: (a) AlCl_3_, CH_2_Cl_2_, –10°C, 16 h; (b) Zinc, I_2_, BrCH_2_CO_2_Et, benzene, reflux, 1 h; (c) (Et)_3_SiH, TFA, BF_3_EtO_2_, CH_2_Cl_2_, reflux, 8 h; KOH/H_2_O, reflux, 8 h; and then PPA, 80 °C, 1 h; (d) NaBH_4_, MeOH, rt, 2 h; (e) 4-hydroxcoumarin, *p*-TsOH, 80 °C, 3 h; (f) HBr, AcOH, reflux, 6 h; (g) 3-(trifluoromethyl)benzyl bromide, sodium hydride, THF, 0 °C, 1 h

*Reagents and conditions*: (a) NaOMe, DMF; (b) BnCu-TMEDA, TMSCl, THF, –78 °C to 30 °C; (c) AlCl_3_, toluene, 90 °C; (d) NaBH_4_, EtOH/THF, rt, then PBr_3_, dichloromethane, 0 °C; (e) HCl(g), 160 °C.

*Reagents and conditions*: (a) 40% KOH; (b) H_3_PO_4_, EtOH; (c) NaBH_4_, MeOH/THF; (d) 4-hydroxycoumarin, *p*-TsOH, 1,2-DCE.

*Reagents and conditions*: (a) pyridine, sodium hydroxide, EtOH.

*Reagents and conditions*: (a) EtOH, AcOH, reflux; (b) (CH_3_CO)_2_CO, –2H_2_O.

*Reagents and conditions*: (a) EtOH reflux 4 h, or microwave irradiation, 15 s.

*Reagents and conditions*: (a) sodium, paraffin, 220-240 °C; (b) sodium, paraffin, 220-230 °C; (c) POCl_3_, reflux.

*Reagents and conditions*: (a) POCl_3_, ZnCl_2_, 75 °C; (b) FeSO_4_, H_2_O, NH_4_OH, 80 °C; (c) NaNO_2_, HCl, and then NaN_3_, 4 °C.

*Reagents and conditions*: (a) KOH, EtOH, 40 °C; SOCl_2_, rt, (+)- or (–)-1,5-dimethyl-4-phenyl-2-imidazolidinone, *n*-BuLi, Ph_3_CH, THF, 0 °C; (b) BnCu-TMEDA-*n*-Bu_2_BOTf, THF, –78 °C to 30 °C; (c) CF_3_SO_3_H, benzene, reflux; (d) NaBH_4_, EtOH/THF (1:1, v/v), then 4-hydroxycoumarin, HCl(g), 160 °C.

*Reagents and conditions*: (a) 10 mol % catalysts **86-88**, dichloromethane, rt.

*Reagents and conditions*: (a) cat-ethylenediammonium diacetate (Tietze base), 5 A molecular sieves, dioxane, 90 °C, screw cap pressure tube; (b) 3N-HCl, SiO_2_, or TFA:H_2_O (19:1, v/v).

**Table 1:** Biological activities for 4-hydroxycoumarin derivatives.

4-Hydroxycoumarin	Biological activity	References
**3**	arthritis, anti-inflammatory anti-cancer, anticoagulant antithrombosis	[33,34] [35,36] [37]
**4**	anticoagulant	[38]
**5**	anticoagulant, teratogenic	[39,40]
**6**	anticoagulant	[41]
**29**	anticoagulant	[42]
**30**	teratogenic	[43]
**40**	vitamin K 2,3-epoxide reductase (VKOR) inhibitors	[21]
**41**	anticoagulant	[44]
**43**	anticoagulant	[45]
**46**	anti-bacterial	[23]
**54**	anticoagulant	[25]
**56**	anticoagulant	[25]
**64**	photoaffinity	[46]
**92**	anticoagulant	[47]
